# Reframing Aging in the Era of Disasters: Strengthening Disaster Preparedness, Response, and Recovery

**DOI:** 10.1093/geroni/igaf122.1972

**Published:** 2025-12-31

**Authors:** Omolola Adepoju, Katie Cherry, Nancy Kusmaul

## Abstract

With the increasing global occurrence of severe weather events and other conflicts, researchers, policymakers, and organizations are focusing on risk, and also strengths and resilience factors of the aging population to mitigate adversity and ease the recovery of older adults. First, Dr. Assaf Suberry will present data on how informal and formal social gatherings enhance coping among older populations with the Israel-Gaza conflict. Second, Piper Bordes will address how positive psychological factors can contribute to post-disaster mental health in younger and older adults using data collected after the 2021 Hurricane Ida. Third, Dr. Katie Cherry will discuss health-related quality of life predictors after exposure to severe weather events. Fourth, Yanjun Don will present FEMA data related to disaster roles, resilience, and recovery. Finally, Dr. Hunter will examine the health impacts of extreme environmental events on older adults from the consideration of the rural-urban continuum. Taken together, these papers provide new behavioral evidence concerning post-disaster health along with new programmatic approaches to disaster mitigation. While age-related changes can make an older person more at risk in a disaster event older adults bring resilience, lived experience, and additional contributions to disaster planning, response, and recovery.

